# Research trend of MRI application for lumbar disc degeneration with low back pain: a bibliometric analysis

**DOI:** 10.3389/fneur.2024.1360091

**Published:** 2024-04-17

**Authors:** Azzam Saeed, Haoyue Shao, Kamal Hezam, Chaoxu Liu, Qiya Zhang, Xiangyu Tang

**Affiliations:** ^1^Department of Radiology, Tongji Hospital Affiliated to Tongji Medical College, Huazhong University of Science and Technology, Wuhan, China; ^2^Nankai University School of Medicine, Tianjin, China; ^3^Department of Orthopedics, Tongji Hospital Affiliated to Tongji Medical College, Huazhong University of Science and Technology, Wuhan, China

**Keywords:** lumbar disc degeneration, low back pain, magnetic resonance imaging, bibliometric analysis, MRI applications

## Abstract

**Background:**

Low Back Pain (LBP) is a pervasive and complex musculoskeletal condition affecting over 80% of the global population. Lumbar Disc Degeneration (LDD) significantly contributes to LBP, and MRI is crucial for its diagnosis and understanding. This study aimes to provide a comprehensive bibliometric analysis of MRI research on LDD with LBP, shedding light on research patterns, collaborations, and potential knowledge gaps.

**Methods:**

A comprehensive online search was conducted in the Scopus database to retrieve published literature on LDD with LBP. Bibliometric analysis was conducted to assess publication patterns, co-authorship networks, keyword co-occurrence, and co-citation analysis within the MRI applications for LDD research domain. Bibliometric analysis tools such as VOSviewer and the R package “bibliometrix” were utilized for quantitative assessments.

**Results:**

A total of 1,619 publications related to MRI and LDD were analyzed. The analysis indicated a consistent annual growth rate of 4.62% in publications related to MRI and lumbar disc degeneration, reflecting a steady increase in research output over the past two decades. The USA, China, and Japan emerged as leading contributors. “SPINE“, “European Spine Journal“, and “Spine Journal” were the most productive journals in this domain. Key research themes identified included lumbar spine, low back pain, and magnetic resonance imaging. Network visualization shows that low back pain and magnetic resonance imaging were the most widely used keywords.

**Conclusion:**

The comprehensive bibliometric analysis of MRI applications for Lumbar Disc Degeneration offers insights into prevailing research patterns, highlights key contributors and journals, and identifies significant research themes. This study provides a foundation for future research efforts and clinical practices in the field, ultimately contributing to the advancement of patient care for individuals suffering from LDD and associated Low Back Pain.

## Introduction

Low back pain (LBP) is a common and multifaceted musculoskeletal condition affecting more than 80% of the global population at various points in their lives (1). Its prevalence ranges from mild discomfort to severe impairment, significantly impacting daily activities, work productivity, and quality of life. This makes it a major concern for individuals and healthcare systems alike (2). One of the primary pathological contributors to low back pain is lumbar disc degeneration (LDD) (3, 4). Lumbar disc degeneration is a process wherein the intervertebral discs, particularly in the lumbar region, undergo structural and biochemical changes. These include loss of disc hydration, reduced disc height, and the formation of tears and fissures in the annulus fibrosus (5). Over time, LDD can lead to reduced shock absorption, increased disc instability, and potentially painful conditions such as disc herniations or stenosis (4). While aging is a natural and primary driver of LDD, other factors such as genetics, obesity, smoking, and occupational hazards can contribute to or accelerate the degenerative process (6–8). Understanding the relationship between LBP and LDD and the early detection of degenerative disc disease (DDD) is crucial for developing effective prevention, diagnostic, and treatment strategies.

Magnetic Resonance Imaging (MRI) has emerged as a critical diagnostic tool in evaluating of lumbar disc degeneration, providing unparalleled insight into the anatomy and pathology of the spinal column (9, 10). Traditional MRI techniques, such as T1-weighted and T2-weighted images, offers a qualitative assessment of lumbar disc morphology, capturing key indicators of degeneration such as disc herniation, reduced disc height, and signal intensity changes (11, 12). Meanwhile, quantitative MRI techniques, including T2 mapping and diffusion-weighted imaging (DTI), offer objective measurements that quantify changes in disc hydration, biochemical composition, and structural integrity (13, 14). These metrics allow clinicians to monitor subtle progression or regression of degeneration over time, offering valuable prognostic information. Furthermore, synthetic MRI presents a newer dimension to spinal imaging, enabling the synthesis of multiple contrast-weighted images from a single acquisition, optimizing scan efficiency and patient comfort (15). Additionally, functional MRI (fMRI) applications are advancing our understanding of pain generation and its association with disc degeneration. By visualizing neural activity, fMRI potentially allows clinicians to discern between painful and non-painful disc degeneration (16). Together, these MRI techniques offer comprehensive insights, shaping decision-making processes and improving patient outcomes in the realm of lumbar disc degeneration.

Bibliometric analysis has emerged as an invaluable tool in the realm of medical research, serving as an analytic technique to quantitatively assess and interpret patterns of publication within scholarly domains (17). By scrutinizing various parameters such as citation counts, co-authorship networks, and keyword co-occurrence, bibliometric studies facilitate an understanding of research topics evolution, knowledge gaps, and influential works and authors recognition within a field (18). A prime advantage of bibliometric methodologies is their ability to map and visualize complex relationships in large datasets, enabling stakeholders to discern trends, alliances, and focal points in research trajectories. The incorporation of bibliometric analysis enriches the medical research landscape. It provides a macroscopic lens through which the progress, collaborations, and innovations in research can be systematically and objectively evaluated, guiding future endeavors and enhancing the breadth and depth of medical knowledge (19).

In light of the burgeoning advancements in medical imaging and the pivotal role of MRI in diagnosing lumbar disc degeneration, there is a compelling need to systematically review and assess the evolving trends in this interdisciplinary domain (20, 21). By harnessing the analytical prowess of bibliometric analysis, this study aims to unravel the trajectories, focal themes, and influential works that have shaped the landscape of MRI applications in lumbar disc degeneration. Leveraging state-of-the-art bibliometric tools such as VOSviewer and the R package, it seeks not only to visualize intricate networks of co-authorship, citations, and keyword interconnections but also to delve into the statistical nuances of the amassed publication data. This comprehensive assessment will illuminate prevailing research patterns, identify potential knowledge gaps, and underscore areas warranting further exploration, ultimately serving to inform and optimize future research efforts and clinical practices in this pivotal medical niche.

## Methods

### Data sources and search strategies

The Scopus database was used to systematically review the literature, with the search timeframe specified from 2000 to 2023. A comprehensive search strategy was developed to find publications related to Lumbar Intervertebral Disk Degeneration (LIVDD). The search incorporated a variety of key terms associated with LIVDD and low back pain, using the following search string: (“Intervertebral Disk Degenerat^*^” OR “intervertebral disk^*^” OR “intervertebral disc^*^” OR “disc herniat^*^” OR “disk herniat^*^” OR “disc disease^*^” OR “disk disease^*^”) AND (“Magnetic Resonance Imaging” OR “MRI”) AND (“lumbar spine”) AND (“back pain” OR “low back pain” AND NOT “without back pain”). This search strategy yielded a total of 1,619 articles, which were subsequently exported as records and references and saved in a CSV file format for further analysis.

### Data collection

Two independent reviewers (A.S and H.S) meticulously verified the data entry and collection process. This included cross-verification of various data points such as titles, keywords, publication dates, authors, affiliated institutions, publishing journals, sum of citations, as well as the countries and regions of the authors. The raw text data, obtained from the Scopus database, were subsequently imported into Microsoft Excel 2019 (Redmond, Washington, USA). For further quantitative and qualitative analysis, the data were also processed using the R package “bibliometrix” and VOSviewer (Leiden University, Leiden, Netherlands).

### Data analysis

The Scopus database was employed to scrutinize various attributes of the publications, encompassing aspects such as the countries and regions of origin, publication timing, author contributions, citation frequency, and H-index. For the bibliometric analysis, a diverse array of platforms was employed, including VOSviewer, Microsoft Excel 2019, and the R package “bibliometrix.” VOSviewer (version 1.6.18), a robust and freely available bibliometric software developed by Nees Jan van Eck and Professor Ludo Waltman at Leiden University, was utilized for co-authorship, co-occurrence, and co-citation analysis (22). Comprehensive examinations were conducted on the retrieved data from three distinct vantage points: authors, organizations, and countries. These were further visualized through network, overlay, and density maps.

The R package “bibliometrix,” initially released in 2016 by Massimo Aria and Corrado Cuccurullo, was another pivotal tool in this study (23). For this analysis, “biblioshiny,” a web interface for bibliometrix, was integrated into RStudio version 1.2.5042. This interface facilitated a multifaceted bibliometric analysis approach, encompassing examinations of conceptual, intellectual, and social structures of knowledge. It yielded unique visual insights through word clouds, thematic maps, and topic dendrograms.

## Results

### Initial findings

As shown in Table 1, this bibliometric analysis provides a comprehensive insight into the research landscape surrounding MRI studies of intervertebral disc degeneration from the years 2000 to 2023. Over this 23-year span, a commendable collection of 1,619 documents was retrieved from 533 distinct sources, which mainly comprised journals but also incorporated books and other materials. This expansive research archive has seen an annual growth rate of 4.62%, showcasing a consistent and burgeoning interest in the domain. The longevity and impact of the documents can be discerned from the average age of the documents, which stands at 8.65 years, and the impressive average citation rate of 20.07 per document. Moreover, when distilled on an annual basis, each document receives approximately 1.824 citations, underscoring the sustained relevance and importance of these studies in the scholarly community.

**Table 1:** Main information about data.

**Description**	**Results**
Timespan	2000: 2023
Sources (Journals, Books, etc)	533
Documents	1,619
Annual growth rate %	4.62
Document average age	8.65
Average citations per doc	20.07
Average citations per year per doc	1.824
References	40,572
Documents per author	0.251
Authors	6,460
Co-authors per doc	5.31
International co-authorships %	15.38
**Document types**	
Article	1,361
Book chapter	5
Conference paper	32
Editorial	12
Letter	29
Note	11
Review	163
Short survey	6

The collaborative nature of the research is evident in the 6,460 authors who have contributed to this corpus of knowledge. And the average number of co-authors per document is 5.31. Further emphasizing the global nature of this research is the fact that 15.38% of these collaborations are international, highlighting cross-border synergies and the universal importance of the topic. In summary, the bibliometric analysis paints a picture of a dynamic, evolving, and collaborative research field where MRI studies of intervertebral disc degeneration are continually pushing the boundaries of understanding, with significant academic contributions and global collaborations at the forefront.

### Annual scientific production and citation

The bibliometric analysis portrays a detailed timeline of the growing interest and endeavors in MRI studies related to intervertebral disc degeneration. From the turn of the millennium in 2000, the scientific community saw a modest initiation with 23 articles being published, a figure that remained consistent through 2001. As the years progressed, a general upward trend in article publication became evident. By 2005, the output had grown to 40 articles, marking an almost two-fold increase from the starting point. The middle of the first decade of the 2000s, particularly between 2005 and 2008, showcased a pronounced surge in research interest, peaking at 57 articles in 2008. Although there was a slight dip in 2007 with 43 articles, the momentum was quickly regained and surpassed in the following years. The period between 2009 and 2012 was particularly prolific, with each year seeing more contributions than the last and peaking at 77 articles in 2012. Interestingly, 2013 witnessed an unexpected decline to 57 articles, reminiscent of the 2008 numbers (Table 2).

**Table 2:** Annual scientific production.

**Year**	**Total publications**	**Total citations**
2000	23	2,388
2001	23	1,172
2002	25	643
2003	33	1,187
2004	36	1,914
2005	40	1,956
2006	51	1,328
2007	43	1,431
2008	57	1,734
2009	63	2,921
2010	67	2,001
2011	71	1,533
2012	77	1,508
2013	57	1,199
2014	71	1,550
2015	75	1,694
2016	82	1,548
2017	92	1,494
2018	89	1,036
2019	101	954
2020	110	588
2021	129	444
2022	139	243
2023	65	32

**Figure 1:**
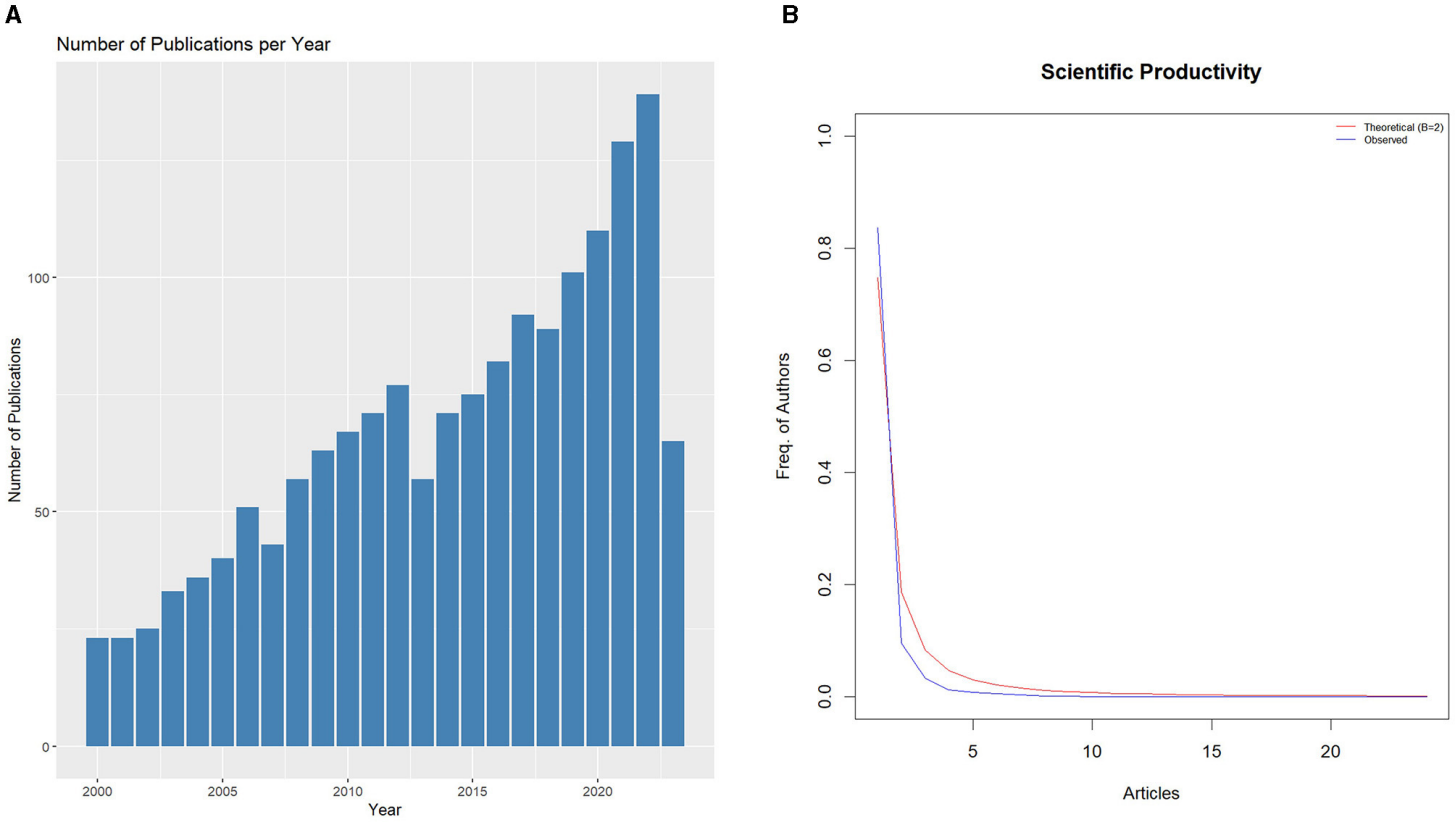
Annual scientific production. **(A)** Number of documents by year. **(B)** Lotka's Law coefficient estimation for describing the frequency of publication by authors.

However, this decline was short-lived, and from 2014 onwards, the community witnessed a robust and almost relentless upward trajectory. By the end of the second decade, the annual scientific output had reached triple digits, with 101 articles in 2019 and a commendable 110 articles in 2020. This rising trend continued into the 2020s, with 2021 and 2022 recording 129 and 139 articles, respectively, as presented in Figures 1A, B. Although 2023 saw a reduction in output with only 65 articles, it is important to consider that the year is potentially still ongoing, and the final figure might increase. The overall annual percentage growth rate, as calculated across these years, stands at 4.62%, underscoring the consistent and gradual expansion of the research landscape. In essence, these results highlight an unwavering commitment and growing scientific intrigue surrounding MRI studies of intervertebral disc degeneration across the span of nearly a quarter-century.

**Figure 2:**
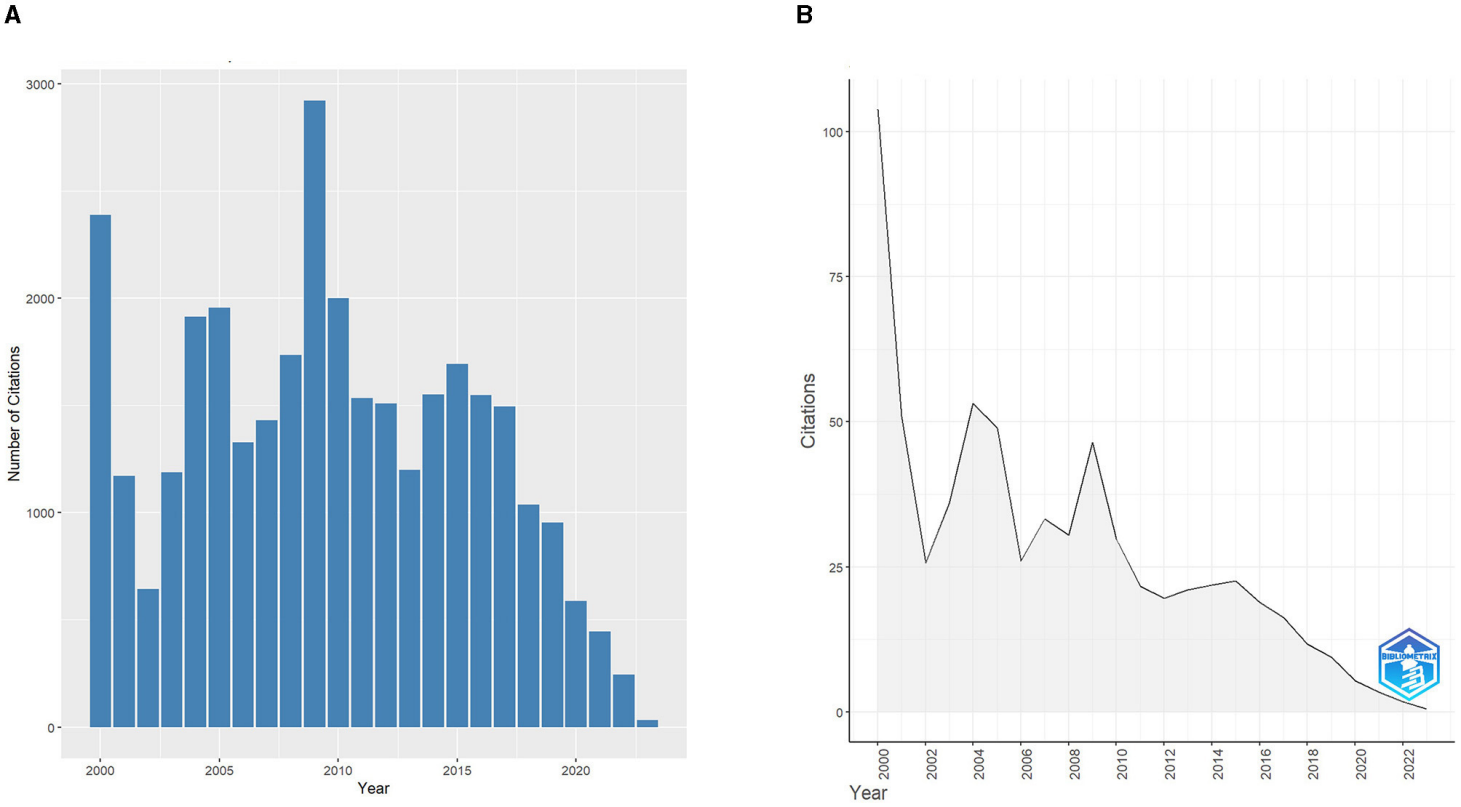
**(A)** Number of citations per year. **(B)** Average citations per year.

These results indicate a notable increase in the total number of publications related to lumbar disc degeneration and associated MRI applications from the year 2000 to 2023. In 2000, there were 23 publications with a total of 2,388 citations, which has escalated significantly to 139 publications with 243 citations in 2022, and 65 publications with 32 citations in 2023. It is observable that while the number of publications has generally shown an upward trend over this period, the number of citations has exhibited variability. For instance, the year 2009 marked a peak in total citations (2,921) for 63 publications, while more recent years, such as 2021 and 2022, have seen substantially fewer citations despite an increase in the number of publications. This trend may suggest a shift in research focus or a delay in the accumulation of citations for newer publications (Figures 2A, B). Overall, the results highlight the growing academic interest in this field, as evidenced by the consistent increase in publications over the years, while the fluctuating citation counts indicate changing impacts and recognitions of these works within the scholarly community.

### Annual trend analysis

**Figure 3:**
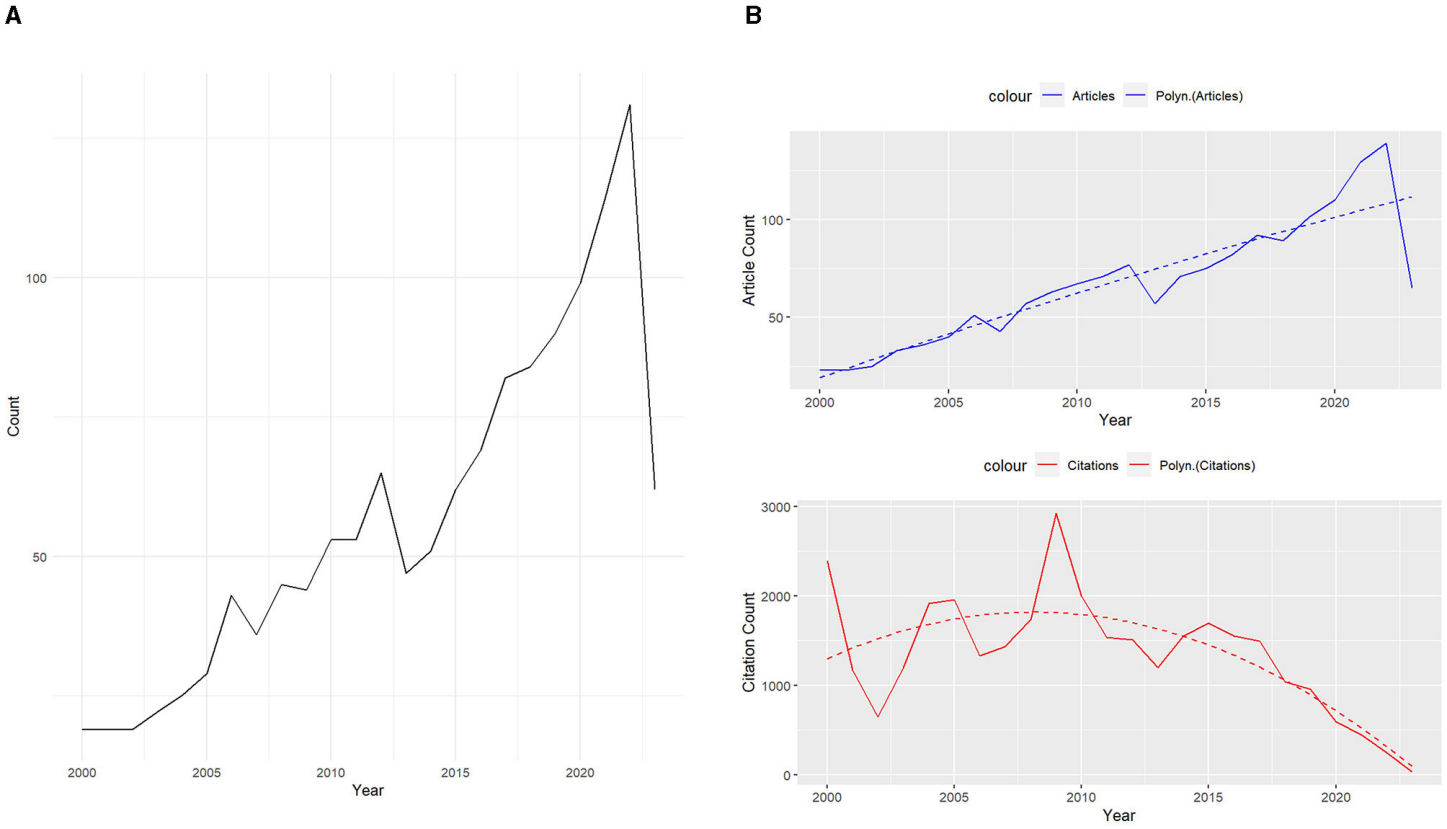
Trends analysis of articles and citations. **(A)** Annual trends for identified topics. **(B)** Annual trends analysis of articles and citations.

The annual trend analysis indicates a generally increasing pattern in both the number of publications and citations related to lumbar disc degeneration and MRI applications (Figure 3A). Starting from the year 2000, with 23 publications and 2,388 citations, there has been a substantial growth in the volume of publications, reaching a peak of 139 publications in 2022. The total number of citations fluctuated over the years, with notable peaks. Despite a few intermittent drops, there is a consistent upward trend in the number of publications over the years, reflecting growing research interest in this field (Figure 3B). In contrast, the total citations per year appear to have peaked in the earlier years of the study period, particularly in 2000 and 2009. From 2018 onward, there is a noticeable decline in the total number of citations, which may be reflective of the more recent nature of these publications and the time needed for subsequent citing of this work (Figure 3C). Nonetheless, the persistent production of new publications suggests that this area of research remains a focal point of interest and is likely to continue evolving as a significant domain within healthcare and medical imaging studies.

### Most productive authors

**Figure 4:**
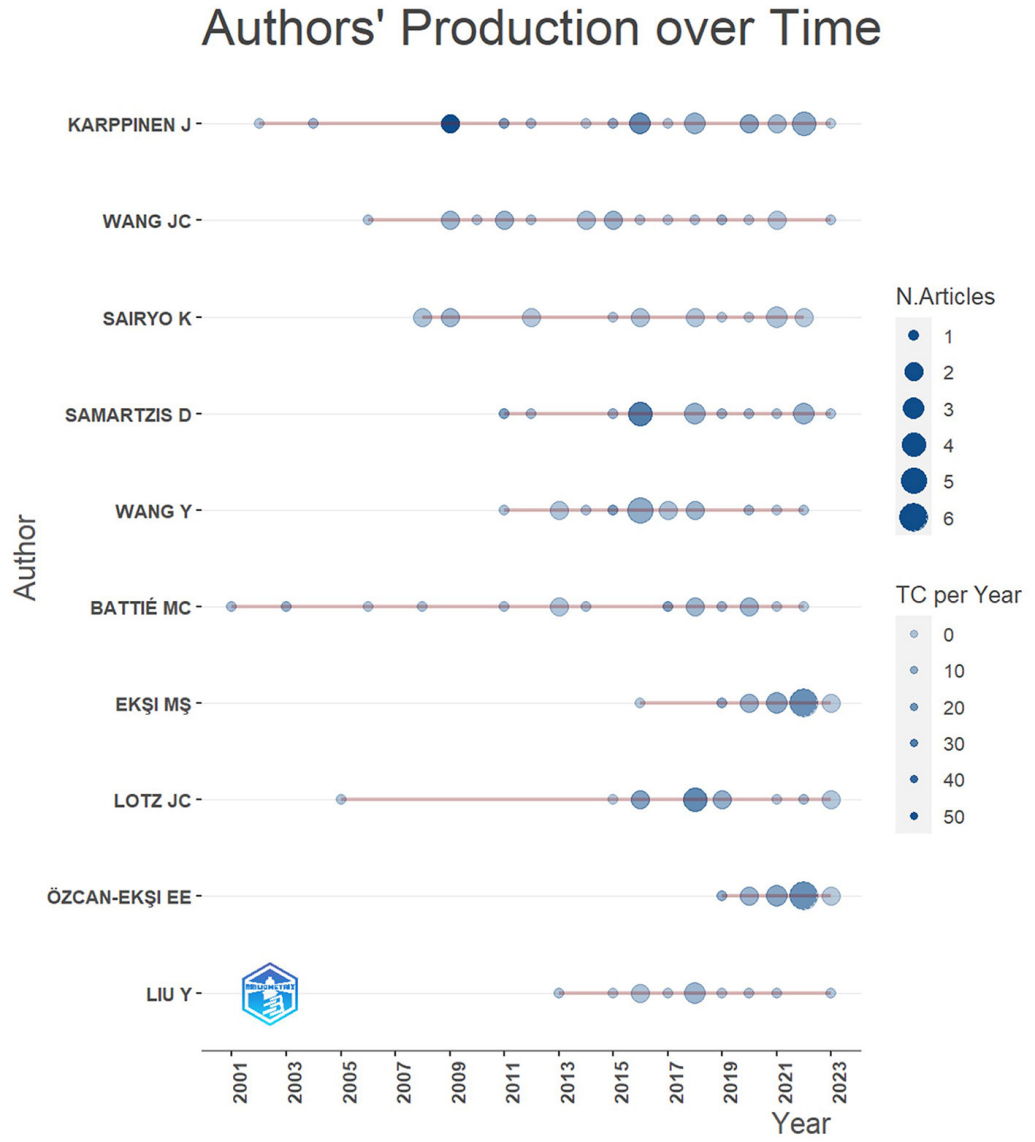
Most productive authors.

The analysis of Figure 4 and Table 3 offer insights into the most prolific contributors to the MRI studies of intervertebral disc degeneration. Topping the list is CHEN S, who has made a commendable contribution with a total of 35 published papers, reinforcing his dominance and expertise in the field. Following closely is Ball JR, who has penned 33 articles, underlining his significant influence and knowledge on the subject. The third spot is occupied by Liu T-F, with a substantial 24 publications to his credit. Natalia F and Andrade JP both share the subsequent position, having contributed 21 articles each, highlighting their parallel prominence in the research domain. Hebelka H, Van Der Heijde D, and Groff MW have also made notable contributions, each with a tally of 20, 20, and 19 articles respectively, reinforcing the depth and breadth of their research endeavors. In summation, these authors, with their prolific outputs, not only epitomize dedication and rigor but also play a pivotal role in shaping the trajectory and narrative of research in this specialized domain.

**Table 3:** Most productive authors.

**No**	**Authors**	**Articles**	**Authors**	**Articles fractionalized**
1	Karppinen J	24	Battié MC	3.56
2	Wang JC	19	Wang Y	3.28
3	Sairyo K	18	Karppinen J	3.21
4	Samartzis D	17	Lee S-H	3.19
5	Wang Y	17	Boden SD	3.12
6	Battié MC	16	Wang JC	2.90
7	Ekşi MS	15	Ekşi MS	2.87
8	Lotz JC	14	Jarvik JG	2.78
9	Özcan-Ekşi EE	14	Özcan-Ekşi EE	2.62
10	Liu Y	12	Videman T	2.41
11	Niinimäki J	12	Sairyo K	2.38
12	Sakai T	12	Jinkins JR	2.25
13	Jensen TS	11	Samartzis D	2.21
14	Lee S-H	10	Le Huec J-C	2.01
15	Luk KDK	10	Benoist M	2.00
16	Chen J	9	Chang Mc	1.92
17	Chen Y	9	Chaudhary V	1.92
18	Cheung KMC	9	Wang Z-X	1.92
19	Espeland A	9	Luk KDK	1.81
20	Hebelka H	9	Feydy A	1.77

Articles fractionalized: fractional authorship measures the extent of an individual author's input across a collection of published papers, under the assumption that each co-author contributes equally to every document.

### Most productive journals

**Figure 5:**
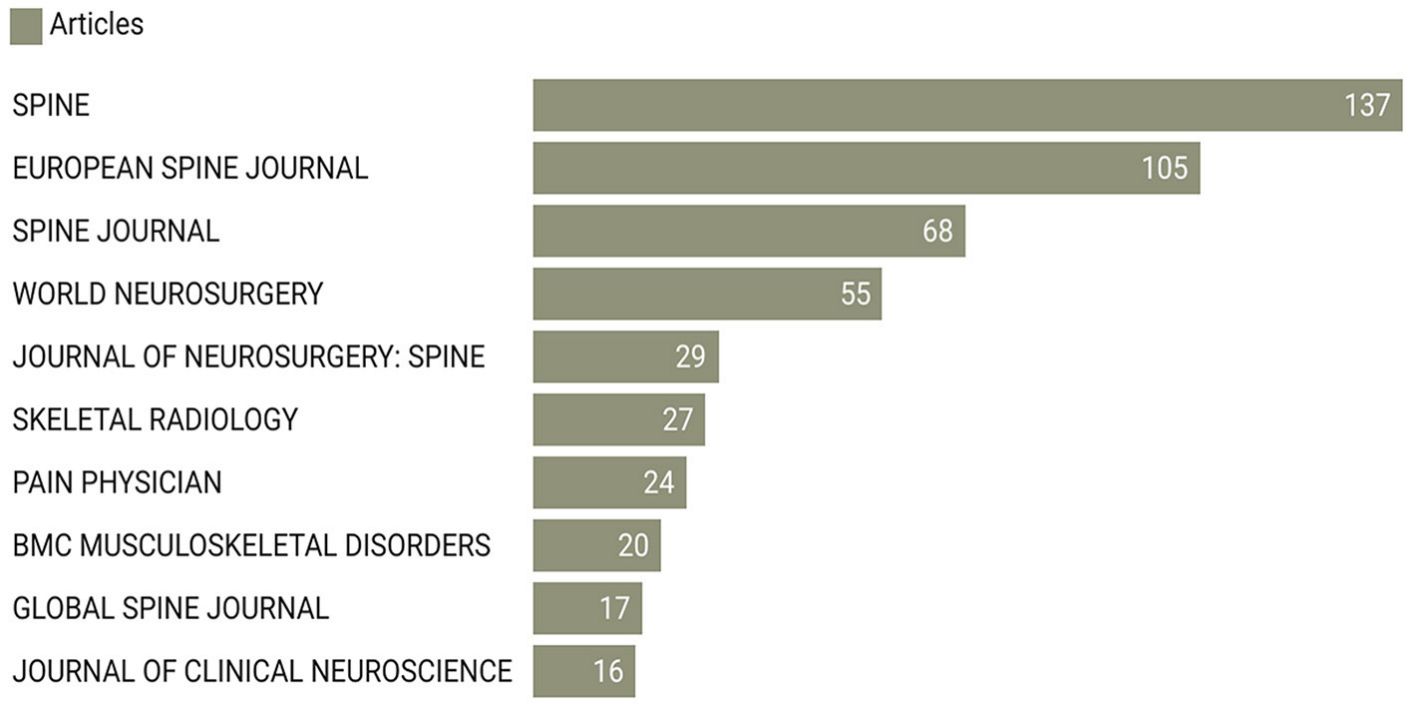
Top 10 journals by number of publications.

This analysis also reveals the foremost journals serving as the primary platforms for disseminating research on MRI studies of intervertebral disc degeneration. As presenting in Figure 5, SPINE is the most productive journal with a substantial 137 articles. Following “Spine” is the “European Spine Journal” with 105 articles. The “Spine Journal” and “World Neurosurgery” are the third and fourth journals with 68 and 55 articles respectively. In essence, these journals represent the epicenters of knowledge and innovation in the study of MRI and intervertebral disc degeneration. Their substantial output signifies their importance in guiding the direction, quality, and evolution of research in this specialized field.

### Most active institutions

**Figure 6:**
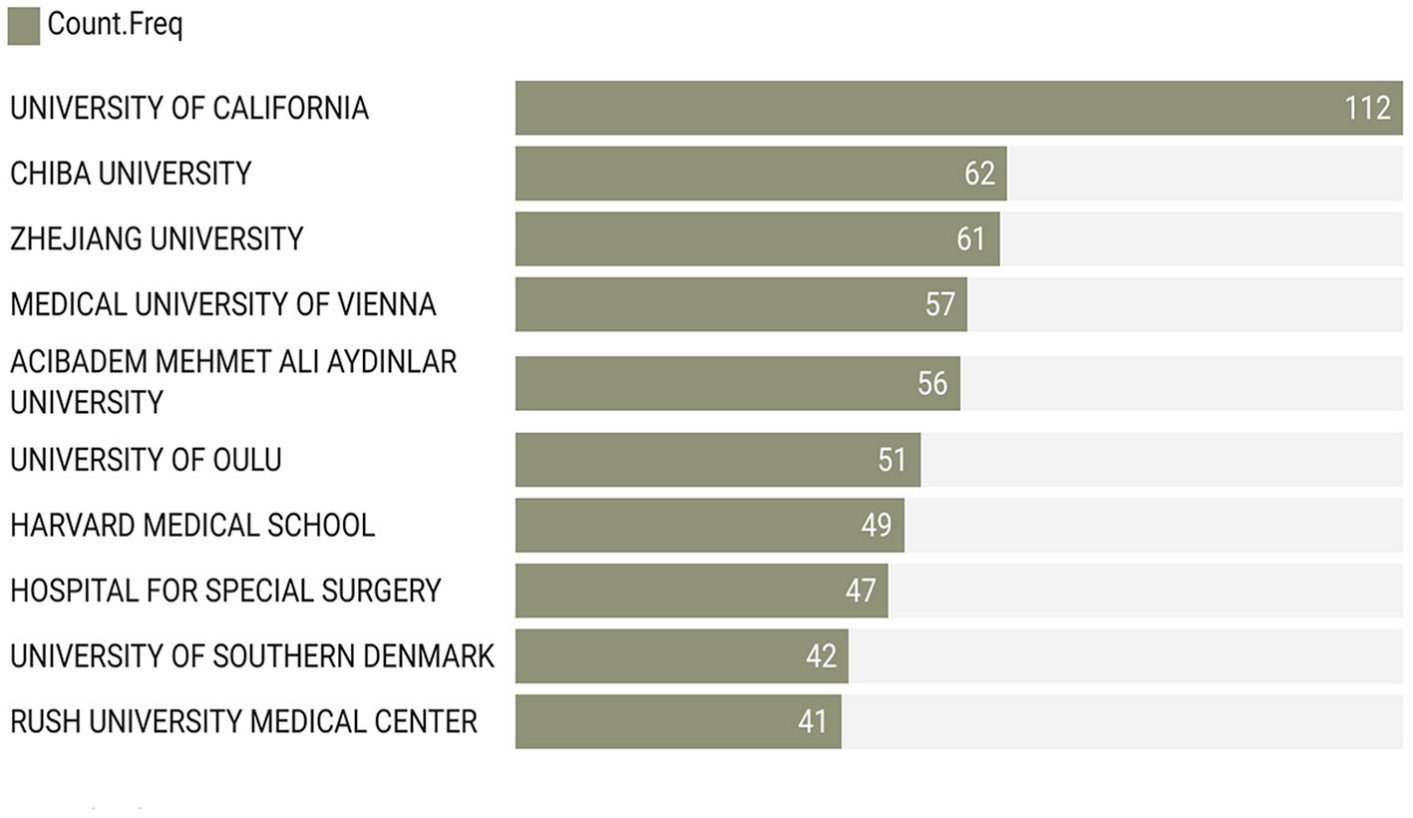
Most active institutions by number of publications.

In the analysis of affiliations contributing to the body of research on lumbar disc degeneration and MRI applications (Figure 6), the University of California stands out as the most prolific contributor, with a total of 112 publications. This is followed by Chiba University in Japan, which has contributed 62 publications, and Zhejiang University in China, with a count of 61 publications. The Medical University of Vienna and Acibadem Mehmet Ali Aydinlar University are also significant contributors, with 57 and 56 publications, respectively. The University of Oulu in Finland has produced 51 publications, while the prestigious Harvard Medical School in the United States has contributed 49 publications. The Hospital for Special Surgery, based in the United States, is credited with 47 publications, while the University of Southern Denmark and Rush University Medical Center, also in the United States, have contributed 42 and 41 publications, respectively. These findings highlight the global nature of research in this field, with significant contributions emanating from institutions across North America, Europe, and Asia.

### Top cited manuscripts

The bibliometric analysis also sheds light on the works that have indelibly impacted the field of MRI studies of intervertebral disc degeneration. Based on Table 4, leading the cadre of influential manuscripts is Luoma K's 2000 work published in 'SPINE', which has garnered a remarkable 900 citations. This underscores the paper's foundational importance and the substantial influence it has exerted over subsequent research endeavors. Cheung KMC's 2009 article, also published in “Spine”, secures the second position with an impressive 634 citations, attesting to its continued relevance and authority in the field. Borenstein DG's 2001 manuscript in “J Bone Jt Surg Ser A” holds the third spot with 336 citations, emphasizing its enduring impact. Following closely, Kjaer P's 2005 “Spine” publication and Kader DF's 2000 piece in “Clin Radiol” have received 332 and 325 citations respectively, reflecting their significant contributions to the scholarly discourse. In summary, these top-cited manuscripts represent cornerstone contributions to the domain, with each playing a pivotal role in sculpting the academic understanding and framework of MRI studies of intervertebral disc degeneration. Their hefty citation counts stand as testament to their foundational importance and lasting impact.

**Table 4:** Top Cited manuscripts per citations.

**No**	**References**	**Title**	**TC**	**TC/Y**	**NTC**
1	Luoma K et al. (24), Spine	Low back pain in relation to lumbar disc degeneration	900	37.5	8.67
2	Cheung et al. (11), Spine	Prevalence and pattern of lumbar magnetic resonance imaging changes in a population study of one thousand forty-three individuals	634	42.3	13.67
3	Borenstein et al. (25), J Bone JT Surg Ser A	The value of magnetic resonance imaging of the lumbar spine to predict low-back pain in asymptomatic subjects: a seven-year follow-up study	336	14.6	6.59
4	Kjaer et al. (26), Spine	Magnetic resonance imaging and low back pain in adults: a diagnostic imaging study of 40-year-old men and women	332	17.5	6.79
5	Kader et al. (27), Clin Radiol	Correlation between the mri changes in the lumbar multifidus muscles and leg pain	325	13.5	3.13
5	Barrey et al. (28), Eur Spine J	Compensatory mechanisms contributing to keep the sagittal balance of the spine	302	27.5	14.36
6	Brinjikji et al. (29), Am J Neuroradiol	MRI findings of disc degeneration are more prevalent in adults with low back pain than in asymptomatic controls: a systematic review and meta-analysis	291	32.3	12.88
7	Carragee et al. (30), Spine	The rates of false-positive lumbar discography in select patients without low back symptoms	283	11.8	2.73
9	Liuke et al. (31), Int J Obes	Disc degeneration of the lumbar spine in relation to overweight	280	14.7	5.73
10	Teraguchi et al. (21), Osteoarthritis Cartilage	Prevalence and distribution of intervertebral disc degeneration over the entire spine in apopulation-based cohort: the wakayama spine study	266	26.6	12.18

TC, Total Citations; TC/Y, Total Citations Per Year; NTC, Normalized Total Citations.

### Corresponding author's countries

**Figure 7:**
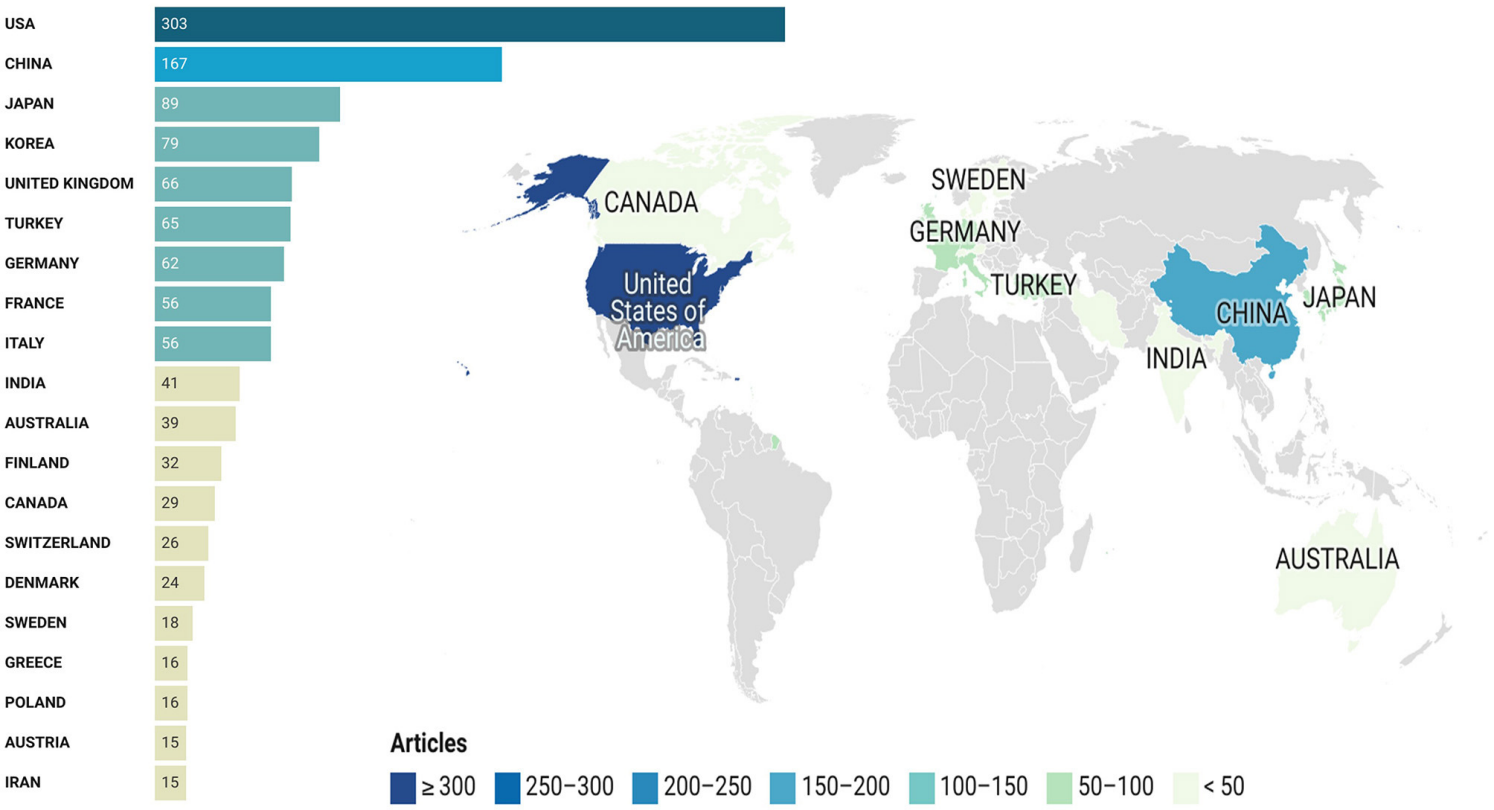
Top prolific corresponding authors' countries.

The geographical distribution of the corresponding authors provides a unique lens into the global contributions to MRI studies of intervertebral disc degeneration, as shown in Figure 7, Table 5. The USA leads the field, with 303 articles, affirming its position as a primary research center in this area. China follows with 167 articles, demonstrating its growing role and dedication to advancing research in MRI studies of intervertebral disc degeneration. Japan, with 89 articles, underscores the significant contribution of the Asian continent to the global research landscape. Korea, with 79 articles, and the United Kingdom, with 66 articles, further highlight the diverse and global nature of this research, emphasizing Europe's and Asia's influential roles. These findings illustrate the collaborative and international effort in enhancing the understanding of intervertebral disc degeneration through MRI studies.

**Table 5:** Corresponding author's countries.

**No**	**Country**	**Articles**	**Freq**	**SCP**	**MCP**	**MCP_Ratio**
1	USA	303	0.2175	262	41	0.1353
2	China	167	0.1199	147	20	0.1198
3	Japan	89	0.0639	79	10	0.1124
4	Korea	79	0.0567	70	9	0.1139
5	United Kingdom	66	0.0474	48	18	0.2727
6	Turkey	65	0.0467	60	5	0.0769
7	Germany	62	0.0445	49	13	0.2097
8	France	56	0.0402	51	5	0.0893
9	Italy	56	0.0402	48	8	0.1429
10	India	41	0.0294	37	4	0.0976

SCP, Single Country Publications; MCP, Multiple Country Publications.

### Most relevant keywords

**Figure 8:**
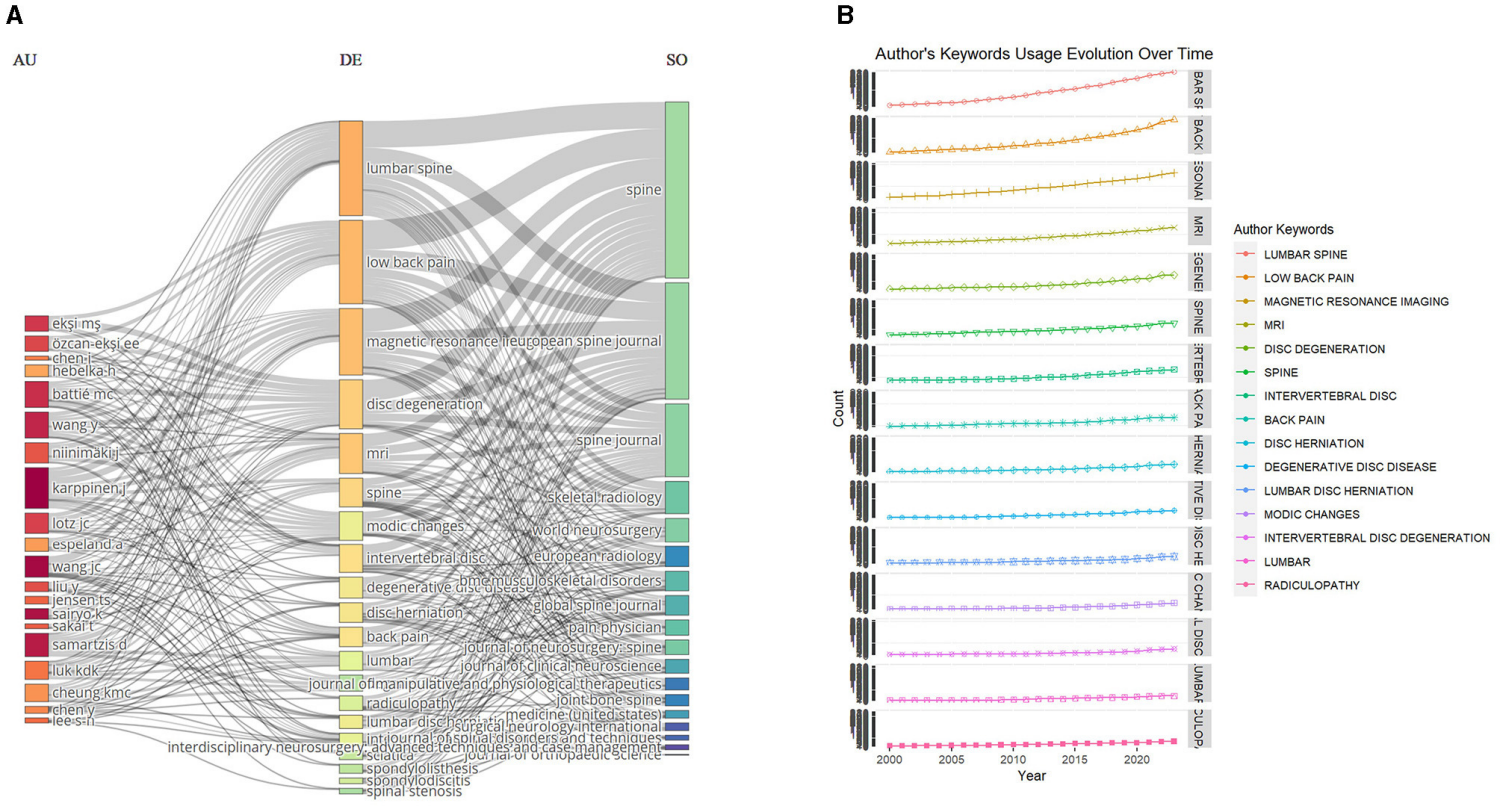
Keywords analysis. **(A)** Three fields plot to visualize the main items of three fields (authors, keywords, journals). **(B)** Author's keywords usage evolution over time.

The observation of the most prevalent keywords offers a clear view of the main themes and areas and focal point in research on MRI studies of intervertebral disc degeneration, as revealed in Figures 8A, B. Topping the list is “Lumbar Spine,” indicating a significant focus on this specific interest of the spine within the research community. The keyword “Low Back Pain” closely follows, reiterating the profound connection between disc degeneration and the manifestation of pain in the lumbar region. Its prominence suggests a vast body of research centered on understanding, diagnosing, and addressing pain stemming from disc issues. “Magnetic Resonance Imaging” and its abbreviated counterpart “MRI” both feature prominently, highlighting the indispensable role of this imaging modality in visualizing and diagnosing disc degenerative conditions. Their prominence underscores MRI's unparalleled capability in providing detailed and non-invasive insights into spinal pathologies, making it a cornerstone in related studies. Lastly, “Disc Degeneration” firmly anchors the list, embodying the core subject of investigation. This keyword's presence reaffirms the academic community's dedication to exploring the various facets, implications, and management strategies associated with degenerative disc conditions. Collectively, these keywords serve as thematic pillars, encapsulating the primary concerns, tools, and areas of exploration within the realm of MRI studies of intervertebral disc degeneration.

### Conceptual structure and factorial map

**Figure 9:**
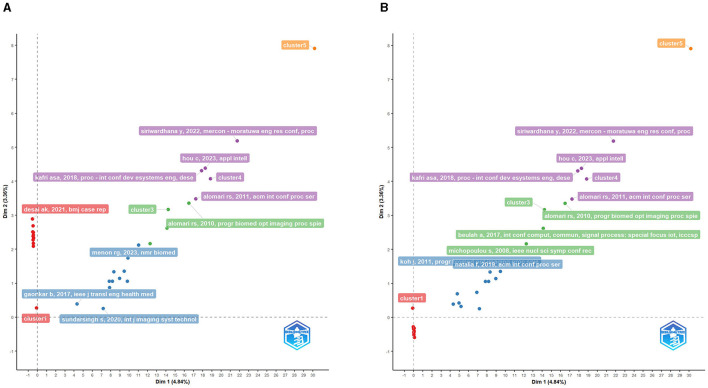
**(A)** Factorial map of the documents with the highest contributes. **(B)** Factorial map of the most cited documents.

The factorial map of the documents with the highest contributions reveals a visual representation of the most influential works in the field of lumbar disc degeneration and MRI applications. The map positions the documents based on their contributions to the field, which is likely measured through various factors such as citation counts, journal impact, and novelty or significance of the findings. Notably, publications from the University of California emerge as significant contributors, reflecting the institution's leading role in advancing research in this domain (Figure 9A). Similarly, the factorial map of the most cited documents provides a visualization of the works that have received the greatest recognition from the scholarly community in this field. These documents are positioned based on their citation counts, offering a clear depiction of the most impactful and authoritative works. The high citation counts associated with these documents underscore their seminal nature and the extent to which they have shaped subsequent research. For instance, works from Chiba University and Zhejiang University are notable for their high citation counts, indicating the significant influence these institutions have within the global research landscape on lumbar disc degeneration and MRI applications, as present in Figure 9B. These factorial maps, collectively, serve as a valuable tool for identifying key players and landmark works in the field, highlighting the central themes that have been the focus of significant scholarly attention, and offering insights into the collaborative and citation networks that underpin this area of medical research.

**Figure 10:**
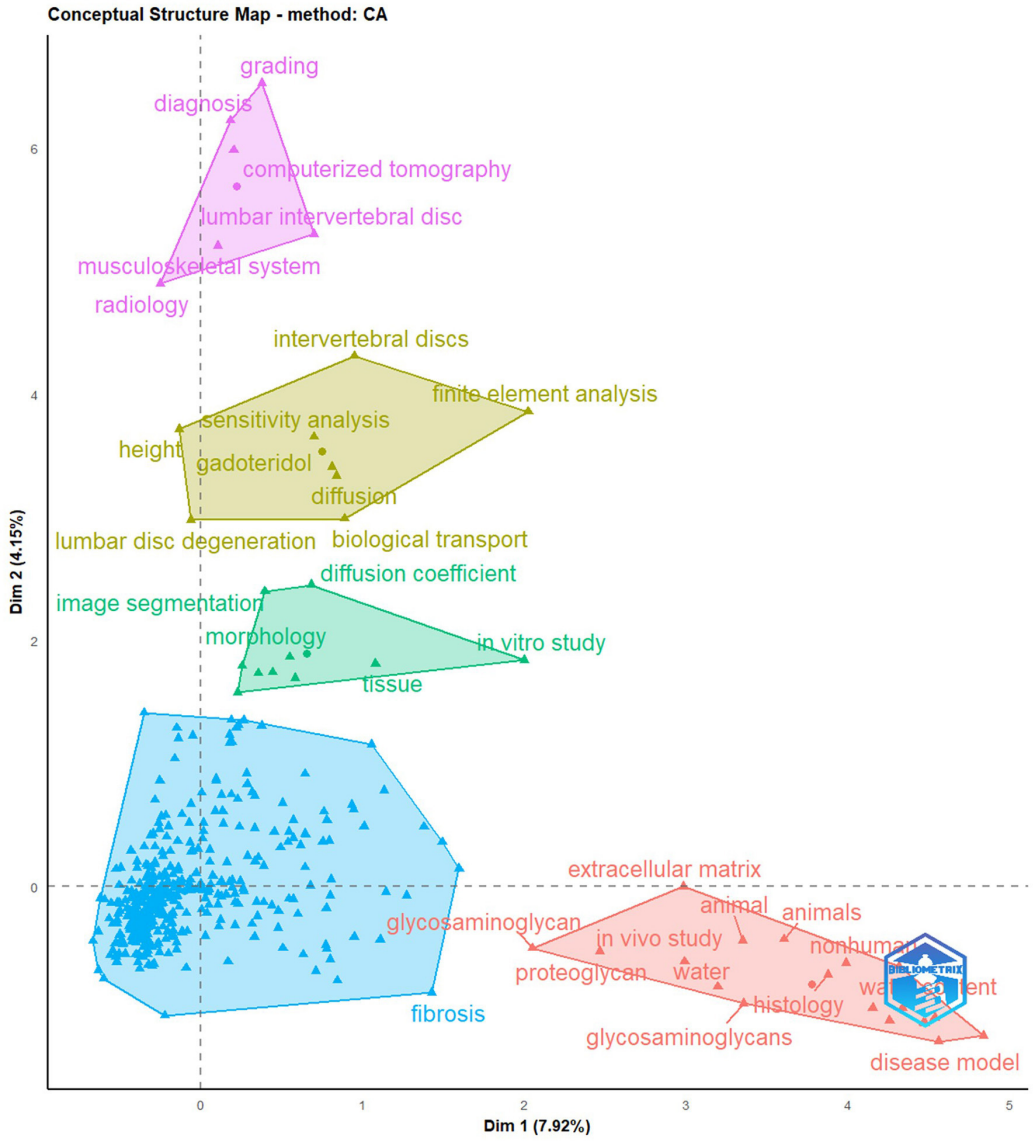
The function conceptual structure map and keyword clusters.

The conceptual structure map in Figure 10, which generated using Correspondence Analysis (CA) method, reveals an intricate web of interrelated concepts and terms among the related publications studying MRI applications in lumbar disc degeneration. The map appears to organize and categorize the research topics into meaningful clusters. In one discernable cluster, we observe concepts related to the diagnostic process, such as “computerized tomography”, “grading”, “diagnosis”, and “radiology”. This cluster likely represents the clinical and radiological methods used to diagnose and assess the severity of lumbar disc degeneration. Another noticeable cluster includes terms like “morphology”, “*in vitro* study”, and “tissue”. This grouping appears to focus on the anatomical and structural aspects of lumbar disc degeneration, indicating a substantial body of research dedicated to understanding the physical changes associated with this condition, often through experimental *in vitro* studies. A third distinct cluster, containing terms like “extracellular matrix”, “animal”, “glycosaminoglycan”, “*in vivo* study”, “proteoglycan”, “water”, “water content”, “histology”, and “disease model”, suggests a focus on the biochemical and molecular aspects of lumbar disc degeneration. This cluster seems to highlight research into the detailed, cellular-level changes that occur during disc degeneration, and how these changes can be detected and quantified using advanced imaging techniques, often through *in vivo* and *in vitro* models. In summary, the conceptual structure map, as depicted through CA method, provides a vivid representation of the multifaceted nature of the research field, emphasizing the diversity and interconnectedness of themes ranging from advanced imaging techniques and anatomical considerations to biochemical analyses and experimental methodologies.

### Visualization map of bibliographic coupling

**Figure 11:**
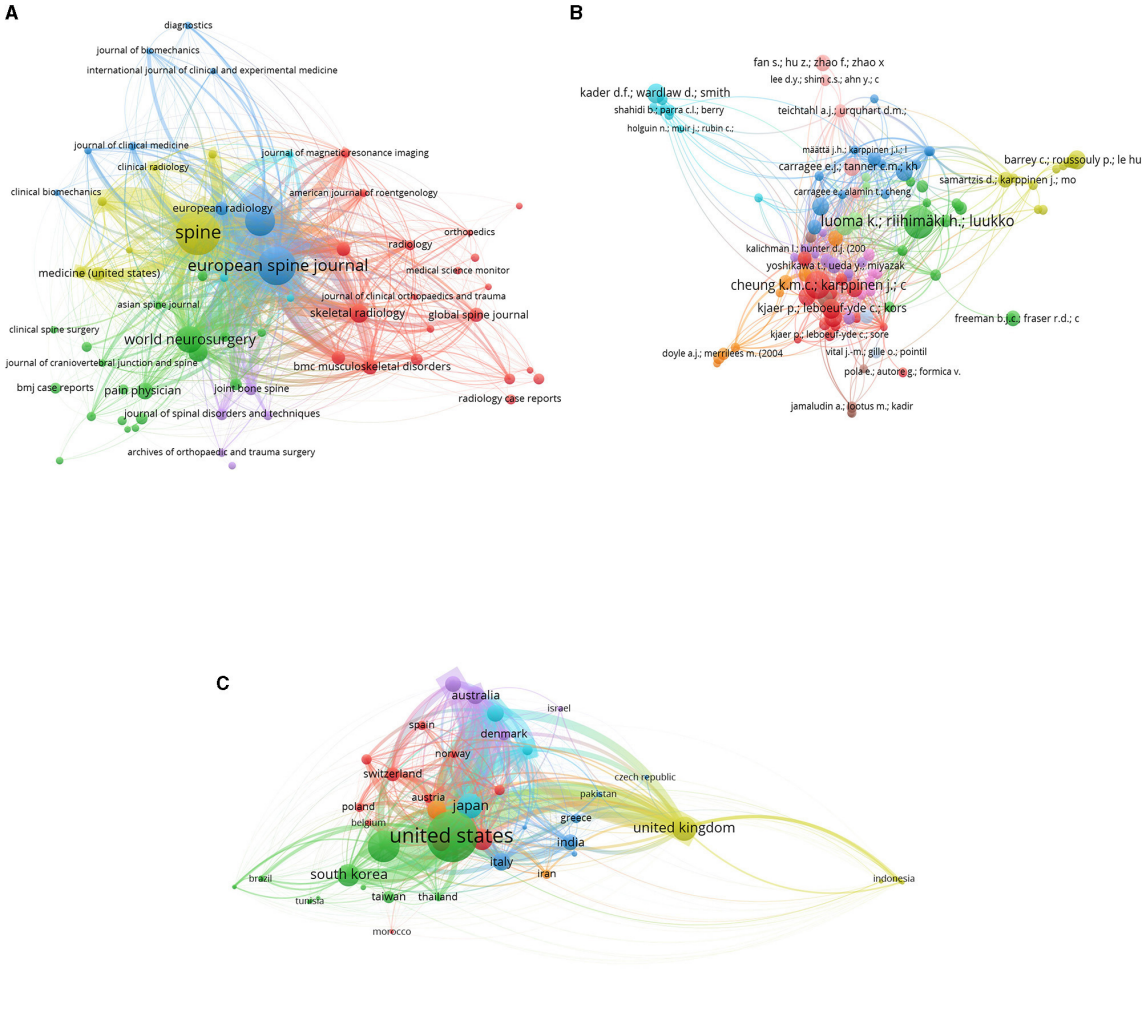
Bibliographic coupling map of Journals **(A)**, documents **(B)** and countries **(C)**.

The visualization map resulting from bibliographic coupling provides a compelling graphical representation of how different research articles are interconnected based on shared references. This method highlights clusters of publications that cite similar foundational works, offering a lens into the thematic commonalities and sub-disciplines within the larger field of study. In such a map, each node typically represents a single publication, with the size of the node often indicative of its prominence or citation count. Lines or edges connecting these nodes signify that they cite a common set of articles. The closer the nodes are to each other, the more significant their shared references, indicating a higher degree of thematic overlap. Upon the results in Figures 11A–C, clusters or groups of closely-knit nodes emerge. These clusters suggest subsets of the research community focusing on MRI studies of intervertebral disc degeneration. The strength and density of connections within these clusters can reveal the most influential or foundational works, which have shaped specific research trajectories. Conversely, nodes that appear more isolated might represent niche areas of research or emerging topics that have yet to gain widespread traction. Furthermore, the overall layout, distribution, and positioning of these clusters can hint at the evolution of research topics over time, indicating shifts in academic interest or the emergence of new paradigms. The visualization map of bibliographic coupling serves as a visual compass, helping researchers navigate the complex web of academic interrelations, understand prevailing research currents, and identify seminal works that act as anchors within the expansive sea of literature.

### Visualization map of keywords

**Figure 12:**
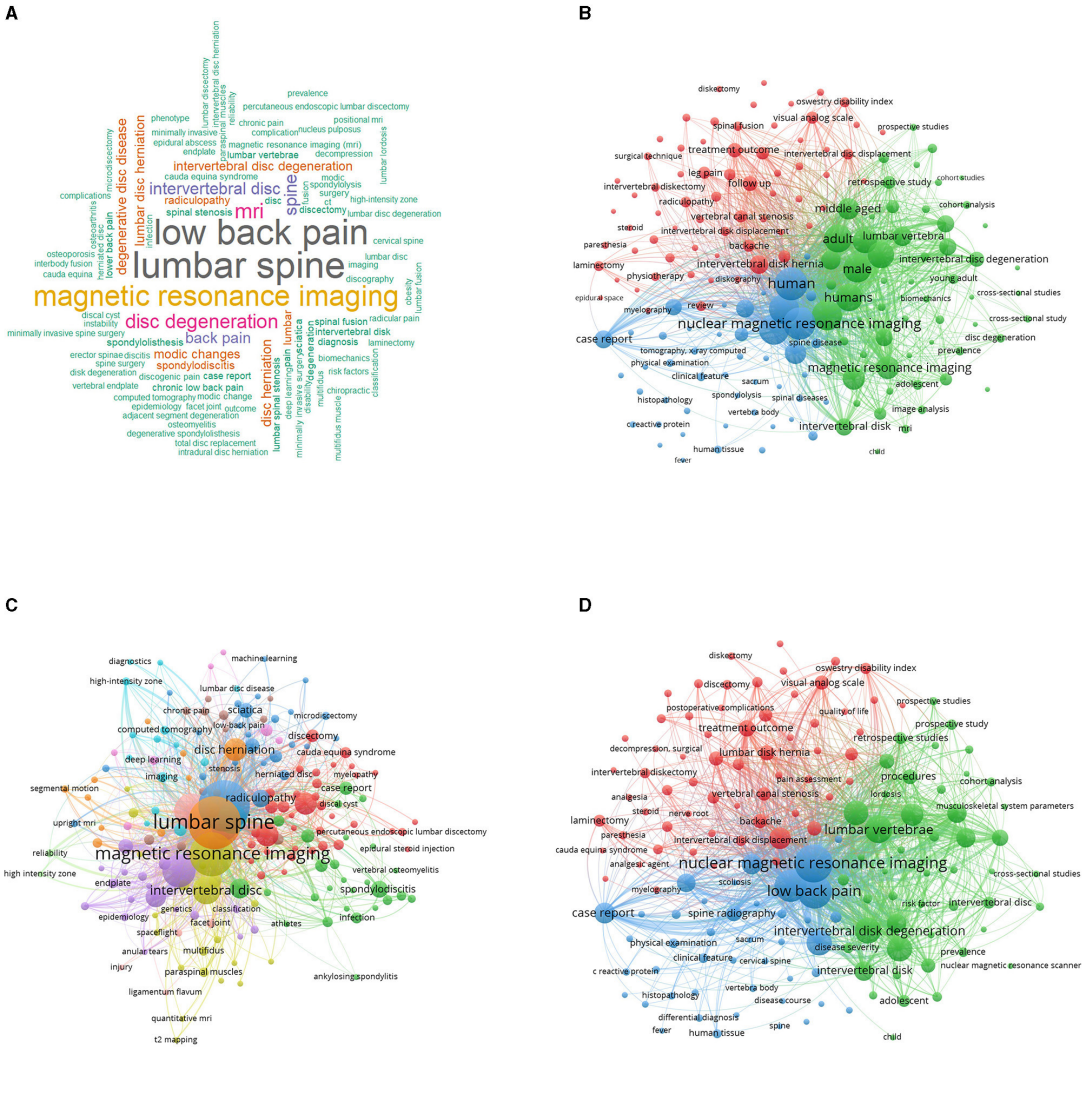
Keywords visualization map for Wordcloud visualization map **(A)**, All keywords map **(B)**, Author keywords map **(C)** and Keywords index map **(D)**.

The visualization map for keyword co-occurrence offers a vivid representation of how certain keywords often appear together within the corpus of research articles. This graphic depiction aids in discerning the interconnected themes and concepts prevalent in the studied domain. In this map, each node usually stands for a specific keyword, with its size often reflecting its frequency or prominence in the dataset. Connecting lines or edges between these nodes signify that those keywords appear in tandem within the same articles. The intensity or thickness of these lines can often denote the strength or frequency of their co-occurrence, and the proximity of nodes to each other suggests a stronger thematic connection. As Figures 12A–D, prominent clusters of tightly interconnected nodes emerge upon examination, suggesting that are closely related or often studied in conjunction. These clusters offer insights into the prevailing research subdomains or areas of concentrated academic interest. Conversely, sparser or more distant nodes might point toward more specialized or emerging keywords, representing niche topics or newer areas of exploration. It's also noteworthy to consider the positioning and interplay between these clusters. Overlapping or closely situated clusters can hint at interdisciplinary research areas or the convergence of themes. Moreover, the visualization can often spotlight central or hub nodes, which act as connecting points for multiple clusters. These hub keywords can be indicative of overarching themes or foundational concepts that underpin various research trajectories. Overall, the keyword co-occurrence visualization map serves as a panoramic lens, illuminating the intricate tapestry of themes, the interplay between concepts, and the foundational pillars anchoring the academic discourse in the field. It enables researchers to grasp the prevailing thematic landscape, identify emerging trends, and recognize areas ripe for further exploration.

### Co-authorship countries visualization

**Figure 13:**
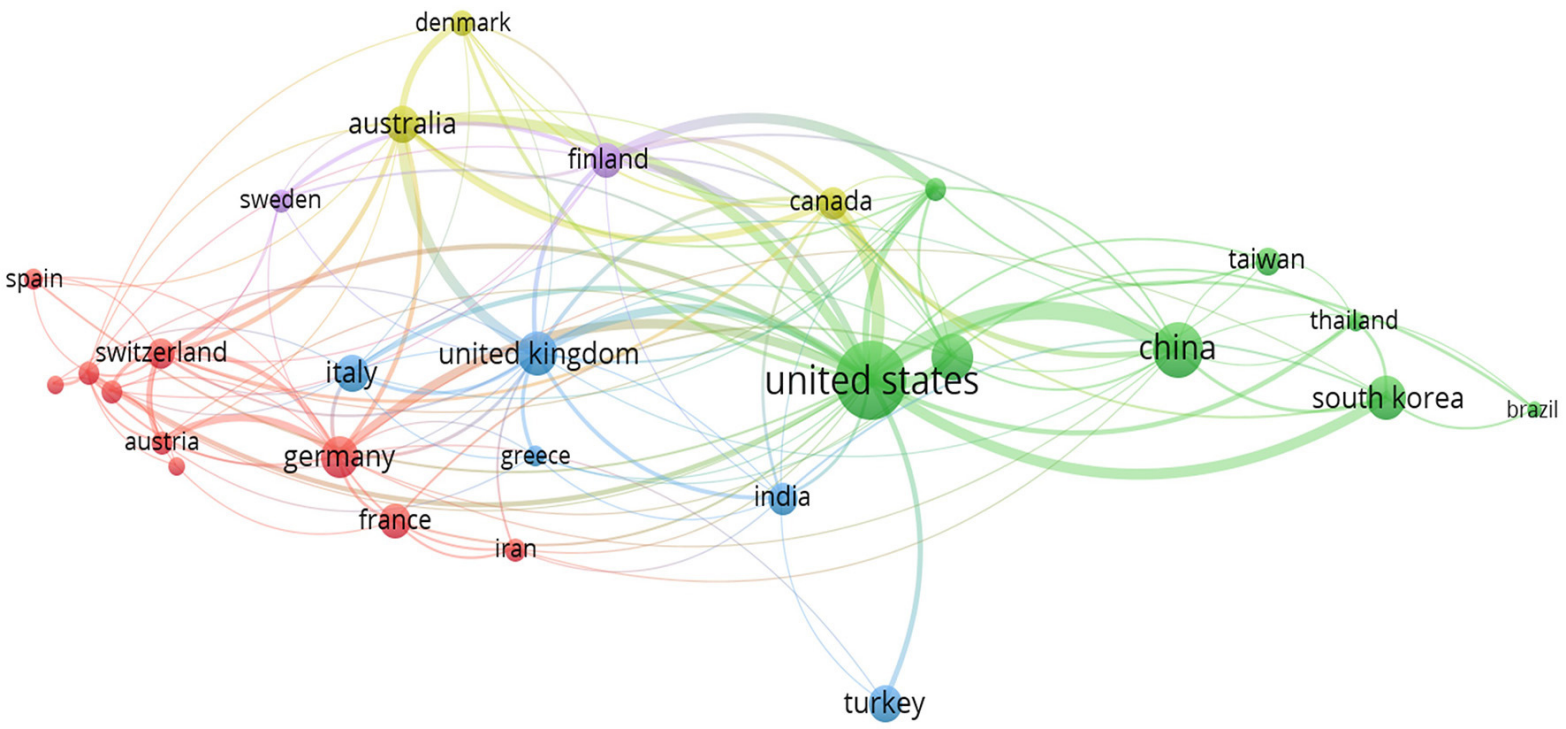
Co-authorship countries visualization map.

The “Co-authorship Countries Visualization” among the related publications delineates several distinct clusters of international collaborations in the field of lumbar disc degeneration research. As presented in Figure 13. Cluster 1 is predominantly composed of Western and Central European countries, including Austria, Belgium, France, Germany, the Netherlands, Norway, Poland, Spain, and Switzerland. Iran is also part of this cluster, suggesting a collaborative link between Europe and Western Asia. This cluster likely indicates a robust network of research collaboration within the European continent, focusing on lumbar disc degeneration. Cluster 2 represents a diverse and global set of collaborations, encompassing countries in Asia—China, Hong Kong, Japan, South Korea, Taiwan, and Thailand as well as Brazil and the United States. This cluster emphasizes the significant level of international collaboration that engages some of the world's largest economies and research hubs in both the East and West. Cluster 3 includes Greece, India, Italy, Turkey, and the United Kingdom. This cluster appears to reflect collaboration between Southern European countries and nations from South Asia and Western Asia, with the United Kingdom likely serving as a significant node in this network. Cluster 4, comprising Australia, Canada, and Denmark, may represent a collaborative research network that spans across continents—from Europe to North America and Oceania. This group suggests a level of collaboration between nations with strong healthcare systems and research infrastructures. Cluster 5, finally, is a Nordic collaboration between Finland and Sweden. This cluster could indicate a close regional cooperation, leveraging shared cultural and systemic approaches to healthcare and research. Overall, the Co-authorship Countries Visualization underscores the truly global and collaborative nature of research efforts aimed at addressing lumbar disc degeneration, revealing intricate networks that span across continents and unite diverse nations in a common scientific pursuit.

### Co-citation visualization map

**Figure 14:**
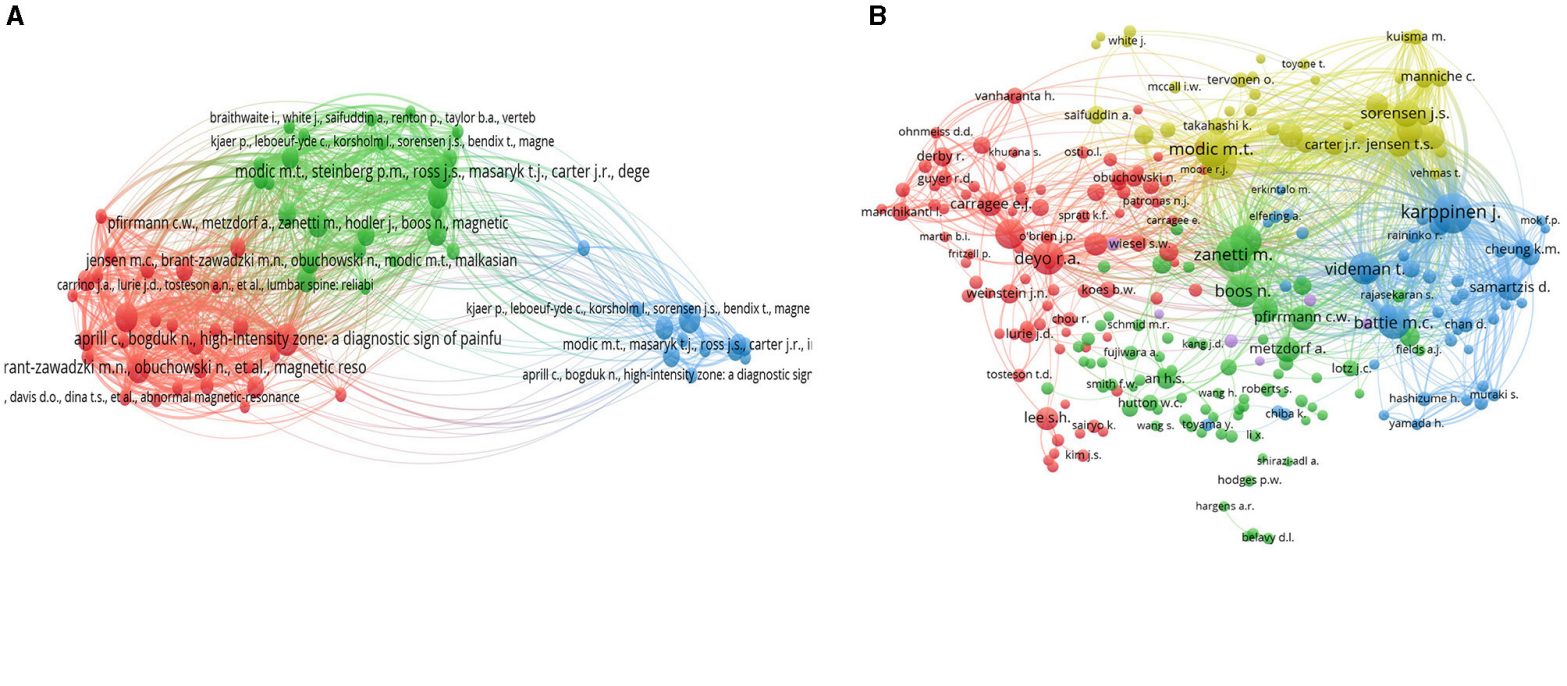
Co-citation visualization map for cited references **(A)** and authors **(B)**.

The co-citation visualization map for cited references among the related publications delineates three distinct clusters of highly cited authors in the field of lumbar disc degeneration research. Cluster 1 features notable scholars such as Adams M.A, Roughley P., Aprill C., Bogduk N., Highboden S.D., Davis D.O., Lurie J.D., and Modic M.T., among others. This group suggests a well-cited community of researchers focused on various aspects of lumbar disc degeneration, potentially including its aging aspects, pathological findings, and radiological evaluations. Modic M.T., a recurrent author in this cluster, is notably associated with the classification of MRI findings in degenerative disc disease. Cluster 2 includes a prominent set of researchers such as Albert H.B., Kjaer P., Andersson G.B., Jensen T.S. and Fairbank J.C. This cluster seems to encompass scholars whose works involve epidemiology, clinical evaluations, and potentially conservative management of lumbar disc degeneration. Albert H.B. and Kjaer P., for example, might be focusing on epidemiological studies and patient management strategies. Cluster 3 involves authors like Aprill C., Bogduk N., Jensen M.C., Kjaer P., Bendix T., Modic M.T., and Pfirrmann C.W., among others. This group likely represents a nexus of scholars dedicated to the radiological and pathological understanding of lumbar disc degeneration. Here again, Modic M.T. and Pfirrmann C.W. are notable figures, often associated with key radiological classifications and imaging standards in disc degeneration. Overall, these clusters represent interconnected groups of researchers whose seminal works are frequently cited together in the literature on lumbar disc degeneration. Their contributions span from imaging and pathophysiological understanding to epidemiological and clinical insights, highlighting the multidisciplinary nature of this research field. These clusters illustrate key thought leaders and influencers in the research community studying lumbar disc degeneration (Figure 14A).

The Co-citation Author Map among the related publications reveals four distinct clusters of highly cited authors in the field of lumbar disc degeneration research. Cluster 1 includes eminent scholars such as Aprill C., Boden S.D., Bogduk N., Carrino J.A., Deyo R.A., and Modic M.T., among others. This group appears to represent a significant collection of authors focused on the clinical, radiological, and pathophysiological aspects of lumbar disc degeneration. Notably, Modic M.T. is a key figure associated with seminal works on MRI findings in degenerative disc disease. Cluster 2 consists of researchers including Albert H.B., Jensen T.S., and Kjaer P. These scholars seem to be engaged in epidemiological studies, patient management strategies, and clinical assessments related to lumbar disc degeneration, suggesting a strong focus on patient-centered research and clinical outcomes. Cluster 3 features authors such as Battie M.C., Cheung K.M., and Karppinen J. This cluster likely represents a group of researchers dedicated to genetic, biomechanical, and environmental factors that influence lumbar disc degeneration, pointing to a more fundamental and mechanistic exploration of the condition. Cluster 4, with scholars such as Adams M.A., Andersson G.B., and Lotz J.C., seems to represent authors who are focused on the biomechanical and morphological aspects of lumbar disc degeneration. Their work likely spans from in-depth analysis of spinal biomechanics to the exploration of degenerative processes at a cellular and molecular level. In summary, these clusters represent interconnected groups of seminal authors whose works are frequently cited together in the literature on lumbar disc degeneration. Their significant contributions range from clinical and radiological studies to epidemiological, biomechanical, and fundamental biological research, highlighting the comprehensive and multidisciplinary nature of this research field (Figure 14B).

## Discussion

This bibliometric analysis conducted on the MRI study of intervertebral disc degeneration offers a comprehensive insight into the research landscape surrounding this topic. Drawing from the vast expanse of data and its subsequent visualization, several salient points and themes emerge that warrant further discussion. The annual scientific production showcases a steady and progressive increase in publications over the years, underscoring the growing significance of MRI in the realm of lumbar disc degeneration. The annual growth rate of 4.62% suggests a consistent and robust interest in the field, hinting at the escalating clinical importance of understanding disc degeneration via MRI. The time trend of publications and citations demonstrates the field's growing recognition and scholarly impact, with a notable increase in the total number of publications over the last two decades. Despite this increase, the recent citation count appears to be decreasing, suggesting that newer publications might not yet have sufficient time to accrue citations or that emerging topics and paradigms are still in the early stages of acknowledgment and validation by the broader scientific community.

The analysis of the most productive authors, journals, and corresponding author's countries offers a snapshot of the main stakeholders driving this research. Notably, authors like Chen S and Ball JR have made significant contributions, emphasizing their pivotal roles in shaping discourse. Geographically, the USA, China, and Japan emerge as leading contributors, highlighting their dominant position in spine and imaging research. Also, specialized journals like Spine and the European Spine Journal are central publishing platforms, reinforcing their authority in spinal research. The top-cited manuscripts serve as a testament to the foundational and groundbreaking research in the domain. The most cited article was published by Luoma K in Spine (24), this study investigated the relationship between low back pain (LBP) and disc degeneration in the lumbar spine. The study found that LBP was associated with signs of disc degeneration, particularly posterior disc bulges, and Occupation was strongly affect the risks of LBP and sciatic pain (24). Another seminal study was published by Cheung K in Spine (11). They conducted a population study involving 1,043 participants to investigate the prevalence and patterns of lumbar changes as detected by MRI. The research provided comprehensive insights into the MRI changes in the lumbar spine region among the study population (11). These manuscripts have garnered significant academic attention, indicating their seminal contributions to the field. An assembly of co-cited papers forms the foundational knowledge in a discipline. When multiple papers are simultaneously cited by one or more articles, it establishes a co-citation linkage. The prominence of keywords like “Lumbar Spine,” “Low Back Pain,” and “Disc Degeneration” in the research corpus underscores the clinical importance of lumbar disc degeneration about back pain and MRI's role in diagnosing and understanding it. The keyword visualization map further emphasizes these thematic intersections, showcasing how central concepts intertwine in the research narrative (32).

This analysis coupling and keyword co-occurrence maps encapsulate the intricate web of interrelated research themes. They spotlight how certain articles and keywords gravitate toward each other, hinting at established sub-domains and potential avenues for interdisciplinary exploration. With the insights gleaned from this bibliometric analysis, researchers can better navigate the voluminous literature, identify pivotal works, and understand prevailing trends. It also illuminates potential research gaps or underexplored areas, paving the way for future investigations.

We also unveil significant trends and patterns in the global research landscape of lumbar disc degeneration, a condition that has major implications for global health given its significant pathological contribution to Low Back Pain. Notably, the co-authorship countries visualization reveals an extensive and intricate network of international collaborations, with Western and Central European countries forming a robust research cluster, possibly facilitated by shared cultural, systemic approaches and proximities within the European Union and neighboring countries. The presence of distinct clusters, such as the strong collaborative network between Asian countries, the Americas, and an apparent bridge created by the United States, underlines the global interest in this research area.

Furthermore, the conceptual structure map elucidates the diverse yet interconnected avenues of research related to lumbar disc degeneration. The map highlights a confluence of methodological approaches from finite element analysis to more clinical aspects like grading and diagnosis—implying a multidisciplinary and concerted effort to tackle the complexities of this condition. The map also reflects a notable emphasis on imaging techniques and biological components (like proteoglycans), indicating a likely trend toward understanding the pathology at a molecular level and leveraging advanced imaging for improved diagnostics and patient management. Understanding the biochemical alterations in the LD are believed to trigger LDD, impacting its biomechanical stability (33). In MRI, the T2WI denotes the transverse relaxation time, a dominant method to diagnose DDD. Despite its prominence, early-stage LDD diagnosis remains challenging due to subtle imaging indicators. Advanced MRI techniques, including DTI, T2 mapping, T1ρ, and now incorporating synthetic and functional MRI, offer enhanced, quantitative evaluations of LDD. For instance, T2 mapping, adeptly detects early-onset LDD and deciphers the biochemical intricacies of LIVD, turning them into quantifiable T2 metrics (34, 35). Evidently, a close alignment exists between T2 mapping outputs and the Pfirrmann grading system, making it a promising imaging standard for a comprehensive LIVD evaluation (36). Contrasting with T2 metrics, T1ρ presents a broader scope to assess LDD, potentially spotting initial degenerative shifts like variations in PG content (37). The focus on DTI's applicability for LDD has expanded lately (38, 39), with DTI offering a detailed 3D perspective of the LVD's structural nuances.

Its core metrics, FA and ADC, enable a granular assessment of water molecule movements, offering insights into the LIVD's structure and function. Notably, the FA and ADC metrics in DTI can shed light on structural changes in nerve roots caused by disc herniations. Synthetic MRI provides multi-parametric imaging (40), allowing clinicians to acquire detailed quantitative information about the biochemical content of the LIVD, particularly the changes in proteoglycan and water content. A primary advantage is its ability to generate T1, T2, and proton density maps, which are instrumental in understanding disc health and degeneration. One study highlighted its utility in the characterization of LIVDs, emphasizing its potential for clinical application in degenerative disc diseases (15). On the other hand, functional MRI, traditionally used in neuroimaging, facilitates the observation of real-time physiological processes in the LDD, helping to elucidate the dynamic interactions within the disc's matrix (16). Physicians engaged in the diagnosis and treatment of LDD should place a heightened emphasis on synergistically integrating the relatively subjective clinical grading assessments with the objective indicators derived from quantitative MRI techniques. Furthermore, future endeavors and continued research in MRI application for LDD should expand and innovate, integrating new sequences for a holistic inquiry and redefine our approach to spine health and disorders.

The analysis is based on the publications available in the Scopus database, and there may be relevant studies that were not included due to database restrictions or language barriers. However, this analysis provides a rich overview of the current state and evolution of this research area. The steady increase in publications, with an annual growth rate of 4.62%, underscores MRI's escalating role in understanding disc degeneration. Interestingly, while publication numbers grow, the recent dip in citation counts suggests emerging topics may still be gaining traction or recognition within the scientific community. The visualization of co-authorship and keyword co-occurrence maps reveals intricate networks and potential interdisciplinary exploration avenues, suggesting a vibrant, collaborative global effort to advance understanding and treatment of lumbar disc degeneration. This analysis not only maps out the landscape but also points out gaps and emerging trends, setting a direction for future research and fostering a deeper understanding of the condition's complexities through advanced MRI techniques.

## Conclusion

The study provides a macroscopic lens through which the progress, collaborations, and innovations in MRI applications for lumbar disc degeneration can be systematically and objectively evaluated. It illuminates prevailing research patterns, identifies potential knowledge gaps, and underscores areas warranting further exploration. This bibliometric analysis paints a portrait of a vibrant, growing, and increasingly collaborative field that is expanding its methodological toolset and deepening its understanding of lumbar disc degeneration. It also sheds light on potential areas for future research, perhaps urging a more detailed exploration of emerging technologies, patient-centered outcomes, and translational research that bridges the gap between basic science and clinical practice.

## Data availability statement

The original contributions presented in the study are included in the article/supplementary material, further inquiries can be directed to the corresponding author.

## Author contributions

AS: Writing – review & editing, Writing – original draft. HS: Writing – review & editing, Writing – original draft. KH: Writing – review & editing, Software, Methodology, Data curation. CL: Writing – review & editing, Supervision, Data curation. QZ: Writing – review & editing, Resources, Investigation, Data curation. XT: Writing – review & editing, Writing – original draft, Supervision, Project administration, Funding acquisition.
